# Detecting immunotherapy-sensitive subtype in gastric cancer using histologic image-based deep learning

**DOI:** 10.1038/s41598-021-02168-4

**Published:** 2021-11-22

**Authors:** Munetoshi Hinata, Tetsuo Ushiku

**Affiliations:** Department of Pathology, The University of Tokyo, 7-3-1 Hongo, Bunkyo-ku, Tokyo, 113-0033 Japan

**Keywords:** Machine learning, Cancer genetics, Gastric cancer

## Abstract

Immune checkpoint inhibitor (ICI) therapy is widely used but effective only in a subset of gastric cancers. Epstein–Barr virus (EBV)-positive and microsatellite instability (MSI) / mismatch repair deficient (dMMR) tumors have been reported to be highly responsive to ICIs. However, detecting these subtypes requires costly techniques, such as immunohistochemistry and molecular testing. In the present study, we constructed a histology-based deep learning model that aimed to screen this immunotherapy-sensitive subgroup efficiently. We processed whole slide images of 408 cases of gastric adenocarcinoma, including 108 EBV, 58 MSI/dMMR, and 242 other subtypes. Many images generated by data augmentation of the learning set were used for training convolutional neural networks to establish an automatic detection platform for EBV and MSI/dMMR subtypes, and the test sets of images were used to verify the learning outcome. Our model detected the subgroup (EBV + MSI/dMMR tumors) with high accuracy in test cases with an area under the curve of 0.947 (0.901–0.992). This result was slightly better than when EBV and MSI/dMMR tumors were detected separately. In an external validation cohort including 244 gastric cancers from The Cancer Genome Atlas database, our model showed a favorable result for detecting the “EBV + MSI/dMMR” subgroup with an AUC of 0.870 (0.809–0.931). In addition, a visualization of the trained neural network highlighted intraepithelial lymphocytosis as the ground for prediction, suggesting that this feature is a discriminative characteristic shared by EBV and MSI/dMMR tumors. Histology-based deep learning models are expected to be used for detecting EBV and MSI/dMMR gastric cancers as economical and less time-consuming alternatives, which may help to effectively stratify patients who respond to ICIs.

## Introduction

According to a recent comprehensive molecular analysis by The Cancer Genome Atlas (TCGA), gastric cancer is categorized into four molecular subtypes: Epstein–Barr virus (EBV)-positive, microsatellite instability (MSI), genomically stable, and chromosomal instable tumors^1^. MSI is caused by underlying defect in the mismatch repair system (dMMR), and immunohistochemistry of mismatch repair proteins is used as a method for MSI determination in gastric cancer^2^. This classification is clinically important because several factors, including prognosis and response to treatments, differ among subtypes^3^. In particular, EBV and MSI/dMMR have been reported to show higher responses to immune checkpoint inhibitors (ICIs)^4^; therefore, identifying these subtypes is important for stratifying patients who respond to ICIs. However, expensive techniques such as immunohistochemistry, in situ hybridization, and polymerase chain reaction are required to determine the subtype, preventing the robust application of molecular subtyping of gastric cancer in practice.

EBV and MSI/dMMR gastric cancers are known to have characteristic histological features. EBV tumors usually show prominent infiltration of lymphocytes into the neoplastic epithelium as well as the stroma and are typically called lymphoepithelioma-like carcinoma or gastric carcinoma with lymphoid stroma^5^. The MSI/dMMR subtype is also known to exhibit abundant lymphocytic infiltration with a predominance of intestinal-type histology and expanding growth patterns^6,7^. These facts indicate that molecular features are reflected in the morphology, at least partly, and that the molecular subtype might be predicted directly from histology. Given that gastric cancer histology comprises broad spectra, it would be difficult for pathologists to reliably detect these subtypes based on histologic images alone.

Deep learning, a method of machine learning, that has been rapidly developed in recent years is being applied to aid in the process of enhancing the broader utilization of histopathology data for subtyping. In particular, deep learning methods using convolutional neural networks (CNNs) have shown excellent results in image recognition^8^. These techniques have also been applied to the analysis of medical images, such as endoscopic, radiographic, and histopathological images. Applications for histopathological images include detection of lymph node metastasis of breast cancer^9^, evaluation of human epidermal growth factor receptor-2 amplification using fluorescence in situ hybridization images^10^, detection of mitotic figures.^11^, and prediction of prognosis in patients^12^.

As for the detection of specific gastric cancer subtypes, Kather et al. showed that deep learning could detect the MSI subtype directly from HE-stained tissue images with moderate accuracy (area under the curve (AUC) = 0.81, internal validation set; 0.69 for external validation set)^13^. They also reported that the presence of EBV infection in gastric cancer could be detected with moderate accuracy (AUC = 0.80, internal validation set, 0.81; external validation set)^14^. The results demonstrate the utility of deep learning in determining molecular subtypes. These reports examined EBV and MSI subtypes independently. However, given that the two subtypes share histological characteristics, it is hypothesized that an analysis combining EBV and MSI/dMMR subtypes into one would lead to more favorable results for detecting the “EBV + MSI/dMMR” subgroup to effectively screen patients who respond to ICIs. In the present study, we trained the deep learning model with a series of whole slide histopathology images of gastric cancer by classifying into “EBV + MSI/dMMR” vs. the others and compared the detection performance with those when classifying EBV and MSI/dMMR independently.

## Materials and methods

### Tissue samples and whole slide images

Formalin-fixed paraffin-embedded gastric adenocarcinoma tissues were retrieved from the archives of the Department of Pathology at the University of Tokyo Hospital (Tokyo, Japan). Tissue samples from surgically resected specimens were used in this study. Tumors with positive staining for EBER-in situ hybridization were defined as EBV (n = 108). Those with deficiency for any of the mismatch repair proteins (MLH1, MSH2, MSH6, and PMS2) by immunohistochemistry were defined as MSI/dMMR (n = 58). Non-EBV and non-MSI/dMMR tumors were defined as the others (n = 242). EBV (n = 42) and MSI/dMMR (n = 58) tumors were screened from 831 consecutive patients who underwent resection between 2005 and 2010. An additional 66 tumors with a diagnosis of EBV were identified from the pathology archive between 1992 and 2018 and included in this study. Tissue microarrays were constructed from these samples and the slides were stained with hematoxylin and eosin (HE). The layers from which tissue microarray cores were obtained varied from case to case. The total numbers of cores from each layer in advanced cases (pT2 or more) were as follows: 52 cores from mucosa, 211 cores from submucosa, 132 cores from muscularis propria, and 23 cores from subserosa (each case contained two cores, and the original histology slide was not available in one case). These tissue microarray slides were digitized using a Nanozoomer 2.0-HT virtual slide scanner (Hamamatsu Photonics, Hamamatsu, Japan), and whole slide images (WSIs) were generated. This study adhered to the tenets of the Declaration of Helsinki, and complies with the STARD reporting guidelines (Supplementary Table S1)^15^. The Research Ethics Committee of the Faculty of Medicine of the University of Tokyo (G3521) approved this study and waived written informed consent because this is a retrospective study using existing pathology slides. Instead, we use an opt out approach to provide participants with an informed choice about participation, although no patient in the cohort for screening used the opt out option.

In addition, gastric cancer cases from TCGA database were used as an external validation cohort^1^. WSIs of HE-stained adenocarcinoma specimens that met the following conditions were included: (1) surgically resected specimens, (2) formalin-fixed paraffin-embedded tissues, and (3) resolutions of WSIs were available. Molecular classification data were obtained from the original paper^1^, and a total of 244 tumors (23 EBV, 44 MSI, and 177 others) from the TCGA cohort were included.

### Image processing

**Figure 1:**
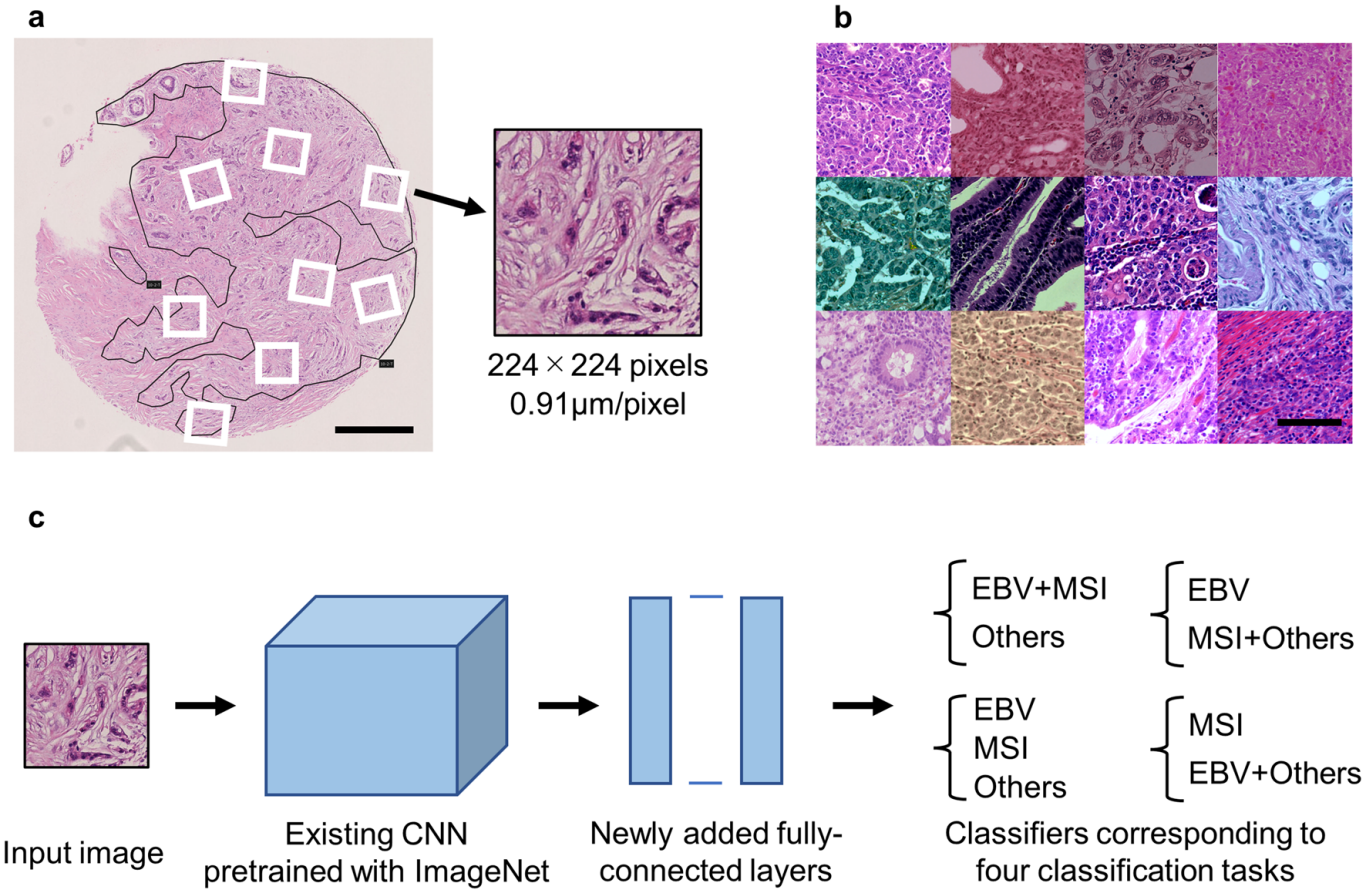
Image processing and the architecture of neural networks. (**a**) Representative image of tissue microarray prepared from cases of gastric cancer. Many small images (224 × 224 pixels) were sampled from annotated tumor areas at random positions and angles. Scale bar: 500 µm. (**b**) Sampled images after data augmentation (random change of color tone and blurring). Scale bar: 100 µm. (**c**) The architecture of neural networks: Fine-tuning of existing CNNs (VGG16, VGG19, ResNet50 and EfficientNetB0) was adopted. Classifiers corresponding to four classification tasks were added on the top. CNN—convolutional neural networks.

The tumor areas of the WSIs were manually annotated by a pathologist using NDP.view2 software (Hamamatsu Photonics, Hamamatsu, Japan). Regarding the cases from University of Tokyo (UTokyo), each case comprised of approximately 8 mm^2^ of tissue, and all areas where viable tumor cells existed were annotated. As for the cases included from the TCGA database, four representative tumor areas per case (total of approximately 16 mm^2^) were annotated because whole tumor areas were too large for the present image processing method. These areas were selected by a pathologist (M.H.), and if morphological heterogeneity existed in the tumor, all different morphological patterns were included as far as possible. A large number of small images (224 × 224 pixels, 0.91 µm/pixel) were sampled from the annotated regions at random positions and angles (Fig. 1a), and these images were used as inputs to the neural networks.

### Data augmentation

For the images used to train the neural networks, data augmentation was performed by changing the color tone and adding blur randomly (Fig. 1b) according to the method described by Tellez et al*.*^16^ (partly modified, see Supplementary Fig. S1 online). In brief, the red, green, and blue (RGB) value was converted to the optical density, and the background was subtracted. Then, the value was deconvoluted into three channels: hematoxylin, eosin, and the remaining. Based on one of the representative cases (one of UTokyo cases with standard staining quality) used in this study, each channel was normalized by multiplying with a coefficient so that the average value would be equal at the patient level. As for TCGA cohort, the coefficients were calculated per each selected area. Consecutively, a random coefficient multiplication was used for each channel, and re-convolution was performed using a random factor. Then, a random background was added, and the optical density was converted into the RGB value. Finally, the brightness, contrast, and saturation were randomly changed. In addition, a Gaussian blur of random intensity was applied to some of these images.

### Deep learning models

CNNs that are pre-trained using ImageNet datasets^17^ were used as the base networks (Fig. 1c). Multiple existing CNNs (VGG16, VGG19^18^, ResNet50^19^, and EfficientNetB0^20^) were prepared to find an appropriate network for this purpose. Fully connected layers were removed from these networks, and new fully connected layers constructed for cancer classification were added to the top. The softmax function (ternary classification) or sigmoid function (binary classification) was used as activation functions of the final output. Each output value corresponds to the probability that a certain image belongs to the class. During training, fine-tuning was performed using the newly prepared datasets. Because a decrease in validation accuracy or an increase in the value of the loss function was observed at some point of training, early stopping was adopted (training was stopped when the average value of the loss function attained the lowest value). Details of the deep learning models and hyperparameters are provided in Supplementary Fig. S2 and Supplementary Table S2 online.

### Construction of training datasets

Patients from UTokyo were randomly divided into five groups at the patient level. The division process was arranged such that the distribution of molecular classification and tumor depth (pT1 or pT2-4) would be uniform. One of these groups was defined as a test dataset and was not used for training purposes. The other four groups were used for training and validation of the neural networks. Three of the four groups were used to train the networks, and the remaining group was used to validate the accuracy. This procedure was repeated four times, rotating the groups (fourfold cross-validation). Finally, all four groups were used to train the networks, and the trained networks were used for subsequent analysis.

In the present study, we have introduced four classification tasks: (1) EBV + MSI vs. others (binary classification of “EBV/MSI” and others), (2) EBV vs. MSI vs. others (ternary classification), 3) EBV vs. MSI + others (binary classification of EBV and “MSI/others”) and 4) MSI vs. EBV + others (binary classification of MSI and “EBV/others”). Image pools (224 × 224 pixels each) were constructed for each task so that the frequency of appearance of each class was uniform (33% each for ternary classification and 50% each for binary classification). For training the neural networks as described above, images were fed from these datasets. The training sets included images with and without data augmentation depending on the purpose of the analysis. However, validation sets and test sets included images only without data augmentation.

### Evaluation of patient-level prediction

**Figure 2:**
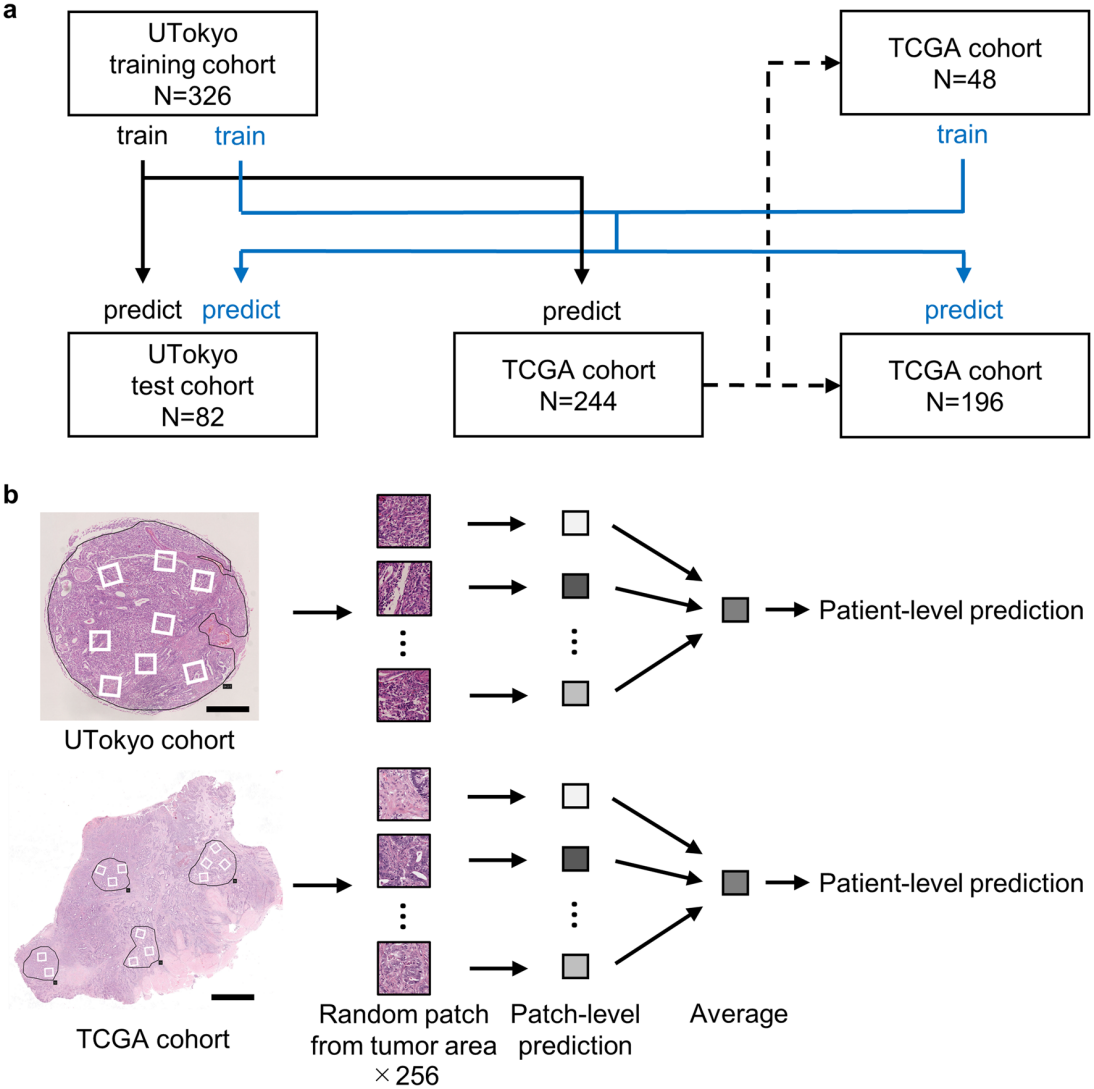
(**a**) The number of patients included in this study. Black solid lines represent the training with only UTokyo cohort, and blue solid lines represent the training with UTokyo cohort and a part of TCGA cohort. (**b**) The flow of patient-level prediction. Scale bar: 0.5 mm (UTokyo cohort), 2.5 mm (TCGA cohort). UTokyo- University of Tokyo, TCGA- The cancer genome atlas.

Two hundred and fifty-six images were randomly selected from the image pools corresponding to each case. The prediction was performed for each image using the trained neural networks, and the result was obtained as an output of the softmax function (ternary classification) or sigmoid function (binary classification). We used a simple method to aggregate these 256 results: calculate the average output value and adopt the class corresponding to the highest value. In the case of binary classification, the receiver operating characteristic (ROC) curve was constructed using the output of the sigmoid function as a variable, and the AUC was evaluated. The number of patients used for training/test, and the flow of patient-level prediction is shown in Fig. 2.

The correlation between tumor mutational burden (TMB) and the classification by our deep learning model was also evaluated for TCGA cohort. TMB was obtained from published data by Thorsson *et al*^21^.

### Visualization of the trained network

The gradient-weighted class activation mapping (Grad-CAM + +)^22^ method was applied to the trained network to visually determine the ground for prediction. The technique of activation maximization^23^ was also applied to investigate the morphological characteristics of the EBV + MSI/dMMR subgroup detected by the neural network.

### Software and hardware

TensorFlow (version 2.3) was used as a deep learning framework^24^. AUCs were calculated using the pROC package^25^ in R, version 4.1.1^26^. Grad-CAM++ and activation maximization were performed using the tf-keras-vis version 0.5.3^27^. Dataset creation, preprocessing steps, training of neural networks, and prediction using neural networks were performed on a machine with Intel Core i7-6900 K processor at 3.20 GHz with 128 GB RAM and four NVIDIA GeForce GTX 1080Ti GPU with 11 GB memory each.

## Results

### Participants

Pathological information of all cohorts included in this study is summarized in Table 1.

**Table 1:** Pathological information of cohorts included in this study. UTokyo, University of Tokyo; TCGA, The cancer genome atlas; EBV, Epstein-Barr virus; MSI, Microsatellite instability; dMMR, mismatch repair deficiency; CIN, chromosomal instable; GS, genomically stable; NA, not available.

Parameters	UTokyo (training)	UTokyo (test)	TCGA
Total number of samples	326	82	244
**Molecular classification**			
EBV	87 (26.7%)	21 (25.6%)	23 (9.4%)
MSI/dMMR	46 (14.1%)	12 (14.6%)	44 (18.0%)
Others	193 (59.2%)	49 (59.8%)	177 (72.5%)
CIN			127 (52.0%)
GS			50 (20.5%)
**Lauren classification for non-EBV and non-MSI/dMMR tumors**			
Intestinal	100 (51.8%)	26 (53.1%)	114 (64.4%)
Diffuse	93 (48.2%)	23 (46.9%)	51 (28.8%)
Mixed			11 (6.2%)
NA			1 (0.6%)
**pT stage**			
pT1	158 (48.5%)	40 (48.8%)	9 (3.7%)
pT2	39 (12.0%)	9 (11.0%)	36 (14.8%)
pT3	66 (20.2%)	17 (20.7%)	142 (58.2%)
pT4	63 (19.3%)	16 (19.5%)	57 (23.4%)

### Comparison of classification accuracy among different CNNs

To find an appropriate CNN architecture, we prepared deep learning models based on different CNNs (VGG16, VGG19, ResNet50, and EfficientNetB0) and compared their validation accuracies. Table 2 shows the validation accuracy of these models in the “EBV + MSI vs. others” tasks with data augmentation. Each value shows the average accuracy (the average ratio of correct prediction for each 224 × 224 pixels image, ranging from 0.0 to 1.0), obtained by fourfold cross-validation. The most accurate model was VGG16-based (0.828), although the average accuracy exceeded 0.8, in either model. The VGG16-base model was used for the subsequent analyses.

**Table 2:** Comparison of classification accuracy between CNNs in the EBV + MSI/others task. Each value shows the average accuracy (the average ratio of correct prediction for each 224 × 224 pixels image, ranging from 0.0 to 1.0), obtained by fourfold cross-validation. The highest accuracy is indicated in bold. CNN, Convolutional neural networks; EBV, Epstein-Barr virus; MSI, Microsatellite instability.

CNN architecture	Validation accuracy
VGG16-based	**0.828**
VGG19-based	0.818
ResNet50-based	0.811
EfficientNetB0-based	0.827

### Accuracy in each classification task

In this study, we trained the VGG16-based CNN using the dataset according to the four classification tasks: (1) EBV + MSI vs. others, (2) EBV vs. MSI vs. others, (3) EBV vs. MSI + others, and (4) MSI vs. EBV + others. Patient-level accuracy was evaluated using test data from the UTokyo cohort (Table 3). The results show the patient-level accuracy of the “EBV + MSI vs. others” task and “EBV vs. MSI vs. others” task for detecting the EBV + MSI/dMMR subgroup. Regarding “EBV vs. MSI + others” and “MSI vs. EBV + others” tasks, the values for detecting EBV subtype and MSI/dMMR subtype are shown, respectively. In the task of “EBV vs. MSI vs. others,” AUC was calculated by subtracting the value of detecting “others” from 1.0. Datasets with data augmentation were used for this examination. Our model accomplished sensitivity 0.879, specificity 0.878, and AUC 0.947 (0.901–0.992) for the “EBV + MSI vs. others” task. In the “EBV vs. MSI vs. others” task, the sensitivity was slightly higher (0.909), however, the specificity and AUC were lower (0.837 and 0.931 [0.876–0.986], respectively) than the “EBV + MSI vs. others” task. In the “EBV vs. MSI + others” task, our model showed particularly high accuracy (AUC = 0.980 [0.956–1.000]), although the accuracy of the “MSI vs. EBV + others” task was moderate (AUC = 0.880 [0.759–1.000]). The sensitivity and specificity did not exceed the result of the “EBV + MSI vs. others” task by the result based on a task combining “EBV vs. MSI + others” and “MSI vs. EBV + others” (i.e., cases predicted as EBV or MSI in either of these two tasks were regarded as EBV + MSI).

**Table 3:** Comparison of patient-level accuracy between classification tasks. The performance in detecting EBV + MSI/dMMR subgroup is shown for “EBV + MSI vs. others” and “EBV vs. MSI vs. others” tasks, whereas the values of “EBV vs. MSI + others” and “MSI vs. EBV + others” tasks are performances for detecting EBV and MSI/dMMR, respectively. “Combination of 3) and 4)” shows the performance for detecting EBV + MSI/dMMR based on the results of 3) and 4) tasks. EBV, Epstein-Barr virus; MSI, Microsatellite instability; dMMR, Mismatch repair deficiency; AUC, Area under the curve; CI, Confidence interval.

Classification task	Sensitivity	Specificity	AUC (95%CI)
(1) EBV + MSI vs. others	0.879 (29/33)	0.878 (43/49)	0.947 (0.901–0.992)
(2) EBV vs. MSI vs. others	0.909 (30/33)	0.837 (41/49)	0.931 (0.876–0.986)
(3) EBV vs. MSI + others	0.857 (18/21)	0.951 (58/61)	0.980 (0.956–1.000)
(4) MSI vs. EBV + others	0.833 (10/12)	0.814 (57/70)	0.880 (0.759–1.000)
Combination of (3) and (4)	0.879 (29/33)	0.735 (36/49)	−

### Validation of accuracy in TCGA cohort

**Figure 3:**
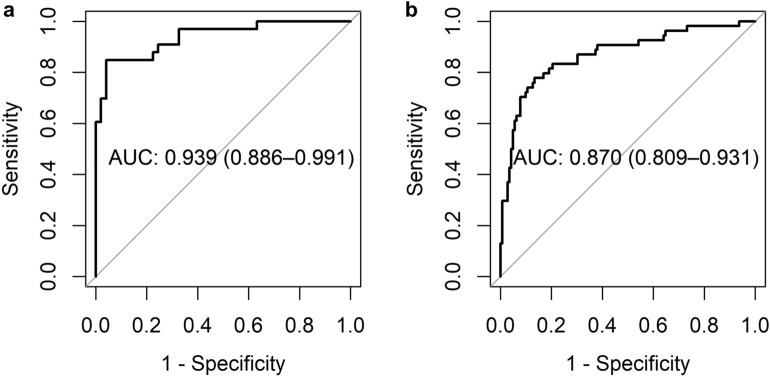
Performance of our model for detecting EBV + MSI/dMMR. ROC curves and AUCs (95%CI) are shown for UTokyo test cases (**a**) and TCGA test cases (**b**). This shows the performance of the model trained with data augmentation and the addition of a part of TCGA cases as training data. AUC- Area under the curve, CI- confidence interval, EBV- Epstein-Barr virus, MSI- microsatellite instability, dMMR- mismatch repair deficiency, ROC-Receiver operating characteristic curve, TCGA- The cancer genome atlas, UTokyo- University of Tokyo.

First, we applied the neural network trained by the UTokyo cohort to an independent cohort from the TCGA database, and the performance of detecting EBV + MSI/dMMR tumors was validated. The results for the “EBV + MSI vs. others” task are shown in Table 4 and Fig. 3. In the TCGA cohort, the detection performance was generally lower than that in the UTokyo cohort. Next, we examined the effect of data augmentation by comparing the results with and without data augmentation. Although a significant difference was not observed in the UTokyo cohort, the accuracy was greatly improved in cases from the TCGA cohort by applying data augmentation (from 0.756 [0.686–0.825] to 0.864 [0.811–0.918] in AUC). In addition, when randomly selected cases from TCGA (20% of all cases) were added to the training data and the remaining 80% of cases were used as test data, a slight improvement in AUC was observed (from 0.864 [0.811–0.918] to 0.870 [0.809–0.931]), and this was the most accurate model for the external validation cohort. Detailed information of the split TCGA cohorts is available in Supplementary Table S3.

**Table 4:** Validation of patient-level accuracy in TCGA cases. Patient-level accuracy is shown for EBV + MSI vs. others task with and without data augmentation. The result when a part of TCGA cases (20% of all cases, randomly selected) was added to the training data and the remaining 80% of cases were used as test data is also shown. The highest AUC is indicated in bold. AUC, Area under the curve; CI, Confidence interval; EBV, Epstein-Barr virus; MSI, Microsatellite instability; TCGA, The cancer genome atlas; UT University of Tokyo.

Data augmentation	Use a part of TCGA cases for training	UT test case			TCGA test case		
		Sensitivity	Specificity	AUC (95%CI)	Sensitivity	Specificity	AUC (95%CI)
−	−	0.848 (28/33)	1.000 (49/49)	0.934 (0.864–1.000)	0.851 (57/67)	0.480 (85/177)	0.756 (0.686–0.825)
−	+	0.818 (27/33)	0.959 (47/49)	0.943 (0.885–1.000)	0.574 (31/54)	0.852 (121/142)	0.800 (0.729–0.871)
+	−	0.879 (29/33)	0.878 (43/49)	**0.947** **(0.901–0.992)**	0.731 (49/67)	0.876 (155/177)	0.864 (0.811–0.918)
+	+	0.848 (28/33)	0.816 (40/49)	0.939 (0.886–0.991)	0.741 (40/54)	0.873 (124/142)	**0.870** **(0.809–0.931)**

We also evaluated the correlation between TMB and classification by our deep learning model for TCGA test cohort (EBV vs. MSI vs. Others task, with data augmentation by random color change and blurring, using a part of TCGA cohort for training). The subgroup classified as MSI/dMMR by our deep learning model showed significantly higher TMB compared to EBV and others subgroups (p < 0.001, Welch’s t-test, Supplementary Fig. S3).

### Explaining the decision of neural network and creation of a “typical EBV + MSI/dMMR” image

**Figure 4:**
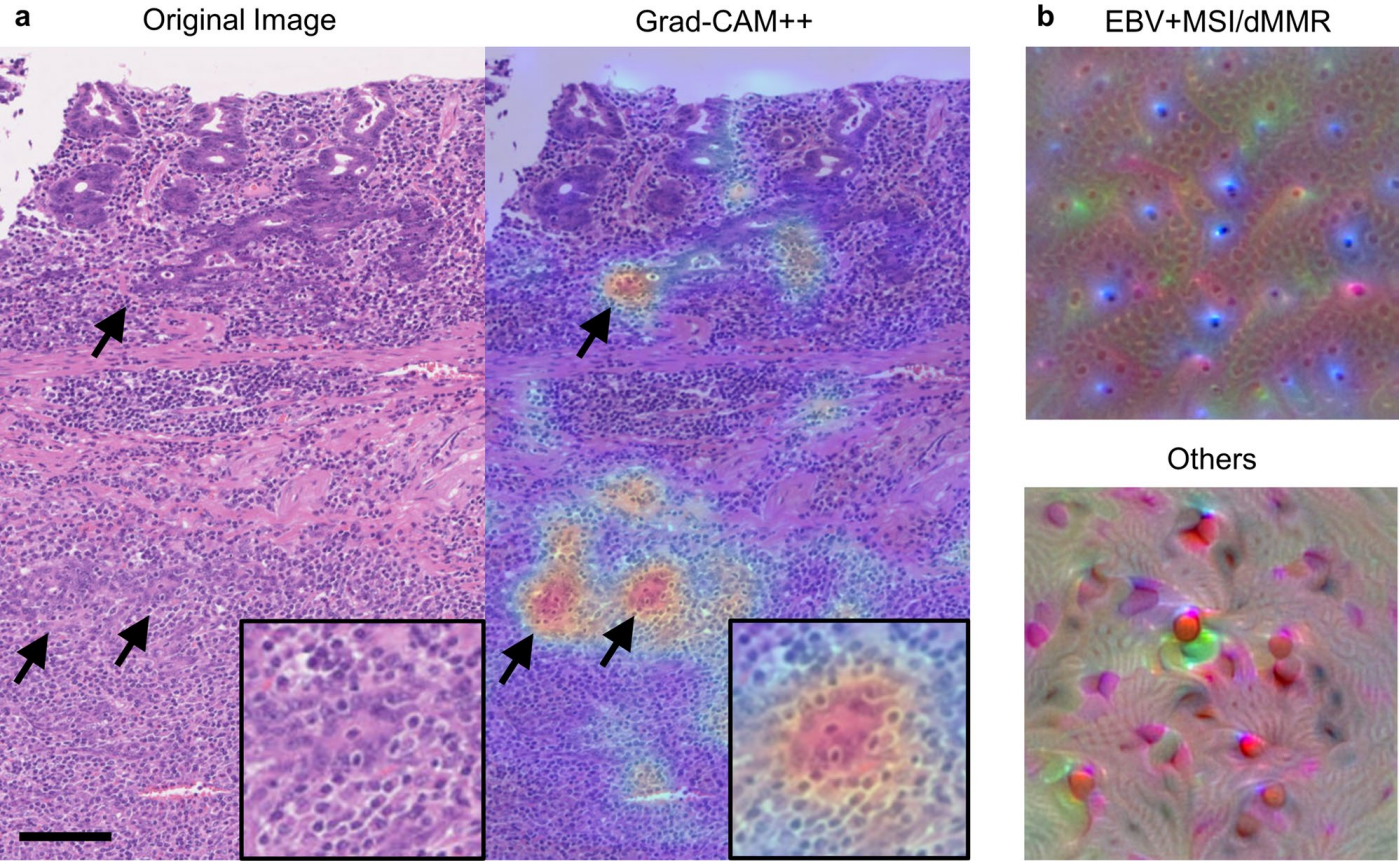
Visualization of the trained network. (**a**) An example of applying Grad- CAM++ to an H&E image (left) of an EBV gastric cancer. Grad- CAM++ (right) highlights the most discriminative area in this image to explain the decision made by the trained neural network (arrow). In a higher magnification (inset), intraepithelial lymphocytosis seems to be the most responsible focus for discriminating EBV + MSI/dMMR from others. Scale bar: 100 µm. (**b**) Created images with the highest probability to be predicted as “EBV + MSI/dMMR” (upper) and “others” (lower) with the method of activation maximization. In the former, the created image seems to represent intraepithelial lymphocytosis. EBV- Epstein-Barr virus, Grad-CAM-gradient-weighted class activation mapping, MSI- microsatellite instability, dMMR- mismatch repair deficiency.

The area of decision-making by the trained CNN was visualized using Grad-CAM++ and activation maximization. In this analysis, we used a trained network that exhibited the highest AUC in the test for the TCGA cohort. Figure 4a shows an example of applying Grad-CAM++ to a typical EBV case. Grad-CAM++ highlighted the most discriminative area in the image to explain the decision made by the trained network. The histologic features of the focus included neoplastic epithelium with intraepithelial lymphocytosis and stromal lymphoplasmacytic infiltration.

Figure 4b shows images created by the activation maximization method that represents an image with the highest probability to be predicted as “EBV + MSI/dMMR” and “others” respectively, by the trained neural network. Notably, the former seemed to represent intraepithelial lymphocytosis and was similar to the focus highlighted by Grad- CAM++ (Fig. 4a).

## Discussion

This study aimed to develop a detector of immunotherapy-sensitive gastric cancer subgroup (EBV + MSI/dMMR) using a histologic image-based deep learning approach. Our model successfully detected the “EBV + MSI/dMMR” subgroup with high accuracy (AUC = 0.947 [0.901–0.992]) for the UTokyo cohort and with moderate accuracy (AUC = 0.870 [0.809–0.931]) for the TCGA cohort. In terms of detection of the “EBV + MSI/dMMR” subgroup, the “EBV + MSI vs. others” task achieved superior performance in comparison to the other tasks that detect EBV and MSI/dMMR tumors independently. In the recent report, Muti et al. showed that robustness of deep learning classifier to detect EBV and MSI in gastric cancer increased when trained on multicenter pooled cohorts^28^. Although to expand cohorts is an important factor to acquire higher accuracy, the effect of combining two similar subtypes into one category has not been investigated. Our observations suggest that combining the two subtypes during the training process could lead to higher detection accuracy, presumably because these subtypes share characteristic histology, such as abundant stromal lymphocytic infiltration and intraepithelial lymphocytosis.

First, we examined the structure of a CNN that is suitable for this purpose. Because it was thought that the amount of training data was too small to train the model from scratch, we adopted fine-tuning of the pre-trained CNNs. In the present study, we constructed CNNs based on four existing models: VGG16, VGG19, ResNet50, and EfficientNetB0, pre-trained using ImageNet datasets. These models have been adopted for histological image analysis in some reports and have achieved some positive results^13,14,29^. The validation accuracy per 224 × 224 pixel images eventually exceeded 0.8 for all models (Table 2), although the model based on VGG16 was slightly more accurate than the others, so we have adopted this model for further analysis.

Next, the accuracies of the different classification tasks were compared. The performance to detect EBV + MSI/dMMR was higher in the “EBV + MSI vs. others” task (sensitivity 0.879, specificity 0.878, AUC 0.947 [0.901–0.992], Table 3) compared to those in the tasks using “EBV vs. MSI vs. others,” and combination of “EBV vs. MSI + others” and “MSI vs. EBV + others.” This result supports the hypothesis that EBV and MSI/dMMR are difficult to distinguish from each other, and the datasets containing these classes separately resulted in “low-quality” data. As for the performance to detect EBV and MSI/dMMR separately, the “EBV vs. MSI + others” task showed high accuracy with an AUC of 0.980 [0.956–1.000], although the “MSI vs. EBV + others” task showed much lower accuracy with an AUC of 0.880 [0.759–1.000]. The number of cases of MSI/dMMR was approximately half that of EBV in this study, and it was considered that the lack of variation in training data led to a decrease in accuracy. Further improvement in accuracy is expected due to the expansion of training data.

We also evaluated the accuracy of the external validation cohort using the TCGA database in order to examine the robustness of our model. The final results were better than those of previous reports^13,14^, although the test data were obtained from different cohorts. In all the patterns examined in this study, the accuracy in cases from TCGA was lower than that in cases from UTokyo. This tendency has been improved by data augmentation by changing color tones and blurring. The difference in staining protocols or digital slide scanners might have affected the results, although other unknown factors might exist. In addition, the accuracy was slightly improved by adding a part of the TCGA cohort for training, suggesting that additional training with the cases of the target institute may be useful for improving robustness.

One of the problems of recent deep learning models is that the decision-making procedure by neural networks is non-transparent, and the predictions are not traceable by humans. Various methods have been proposed to solve the black boxes of deep-learning models. In this study, we adopted Grad- CAM++and activation maximization. Grad- CAM++highlights the areas on which the neural network focuses for decision-making. Interestingly, we found that the network tends to focus on particular areas of an image and that the discriminative focus includes intraepithelial lymphocytosis, a feature characteristic of lymphoepithelioma-like carcinoma. Such histology is considered to be a common characteristic of EBV and MSI/dMMR subtypes, and it is easy for pathologists to understand the prediction results. The images generated by the method of activation maximization represent the most like “EBV + MSI/dMMR” tumor or the most like “others” tumor images for the CNN. Interestingly, the small round structure noted in the “EBV + MSI/dMMR” image can be regarded as intraepithelial and stromal lymphoplasmacytic infiltration, which highlights the features characteristic of EBV and MSI/dMMR. In the “others” image, the wavy structure similar to the desmoplastic pattern of cancer stroma is observed, which was relatively uncommon in EBV and MSI/dMMR tumors. These results emphasize on intraepithelial lymphocytosis as a characteristic of EBV and MSI/dMMR tumors, whereas prominent desmoplastic reaction is unlikely to be a feature of these tumors.

In the clinical context, EBV and MSI/dMMR are different from other subtypes with regard to prognosis and response to treatment. For example, EBV and MSI/dMMR have been reported to have a better prognosis than the other subtypes^30,31^. The frequency of lymphovascular invasion and lymph node metastasis is also low in EBV, which could lead to the expansion of the indications for endoscopic resection^5^. EBV and MSI/dMMR are also known to be sensitive to immune checkpoint inhibitors^4^. These facts suggest the clinical importance of the molecular classification of gastric cancer, especially for identifying EBV and MSI/dMMR subtypes. Deep learning-based subtype detection methods require only HE-stained tissue images that are available from not only digital slide scanners but also digital cameras, most tablet computers or smartphones. The images can be transferred to the detection system through the Internet. Therefore, if an online-based system is constructed, each institute does not have to arrange for the expensive digital slide scanners and deep learning machines. This could contribute to determining clinical strategies owing to their time-efficiency and economical nature, considering that it is difficult for many pathology laboratories to perform molecular tests to detect these subtypes in daily practice as a routine.

There are some limitations in this study. First, we used tissue microarray for UTokyo cohort, and adopted manual annotation of representative tumor areas for TCGA cohort. Given the heterogeneity of tumor tissue, this can be a source of bias compared to using whole tissue slides to prepare datasets. Second, we used manual annotation by a pathologist to specify the tumor area, which can be an obstacle in broad application of this method. Recently, some weakly supervised methods (for example, attention-based deep multiple instance learning^32^) had been developed in the area of deep learning, and these methods might contribute to omitting the process of manual annotation. Finally, our deep learning model developed in this study aims to detect either EBV or MSI gastric cancers, a surrogate marker for response to ICIs. However, it would be more important to develop an algorithm to directly detect responders to ICIs by using a cohort including responders and non-responders in the real-world setting.

In this study, the detection accuracy in the external validation cohort improved by data augmentation and by using a part of the target cohort for training. However, the accuracy was still lower than that of the internal validation cohort. For the wider application of this method, the difference of accuracy between cohorts is one of the problems to be solved in the future.

In conclusion, our deep learning model succeeded in detecting immunotherapy-sensitive gastric cancer subtypes from histological images with high accuracy. It is expected that this method would widen the screening of EBV and MSI/dMMR subtypes to provide more appropriate therapeutic strategies for gastric cancer patients worldwide at a lower cost and in a shorter time than the conventional methods.

## Supplementary Information

Supplementary Information.

## Data Availability

All source codes to process WSIs, and to train and assess our deep learning classifiers are publicly available on GitHub (https://github.com/fircothep262/ebv-msi-detection-by-dl) and Zenodo (https://doi.org/10.5281/zenodo.5552137). The datasets generated during and/or analyzed during the current study are available from the corresponding author on reasonable request.

## References

[1] Cancer Genome Atlas Research Network (2014). Comprehensive molecular characterization of gastric adenocarcinoma. Nature.

[2] Puliga E, Corso S, Pietrantonio F, Giordano S (2021). Microsatellite instability in Gastric Cancer: Between lights and shadows. Cancer Treat Rev..

[3] Pereira MA (2018). Clinicopathological and prognostic features of Epstein-Barr virus infection, microsatellite instability, and PD-L1 expression in gastric cancer. J. Surg. Oncol..

[4] Kelly RJ (2017). Immunotherapy for Esophageal and Gastric Cancer. Am. Soc. Clin. Oncol. Educ. Book.

[5] Fukayama M (2020). Thirty years of Epstein-Barr virus-associated gastric carcinoma. Virchows Arch..

[6] Grogg KL, Lohse CM, Pankratz VS, Halling KC, Smyrk TC (2003). Lymphocyte-rich gastric cancer: associations with Epstein-Barr virus, microsatellite instability, histology, and survival. Mod. Pathol..

[7] Arai T (2013). Frequent microsatellite instability in papillary and solid-type, poorly differentiated adenocarcinomas of the stomach. Gastric Cancer.

[8] Krizhevsky A, Sutskever I, Hinton GE (2012). Imagenet classification with deep convolutional neural networks. Adv. Neural Inf. Process. Syst..

[9] Ehteshami Bejnordi B (2017). Diagnostic Assessment of Deep Learning Algorithms for Detection of Lymph Node Metastases in Women with Breast Cancer. JAMA.

[10] Zakrzewski F (2019). Automated detection of the HER2 gene amplification status in Fluorescence in situ hybridization images for the diagnostics of cancer tissues. Sci. Rep..

[11] Balkenhol MCA (2019). Deep learning assisted mitotic counting for breast cancer. Lab. Invest..

[12] Wang X (2021). Predicting gastric cancer outcome from resected lymph node histopathology images using deep learning. Nat. Commun..

[13] Kather JN (2019). Deep learning can predict microsatellite instability directly from histology in gastrointestinal cancer. Nat. Med..

[14] Kather JN (2019). Deep learning detects virus presence in cancer histology. Preprint at.

[15] Bossuyt PM (2015). STARD 2015: an updated list of essential items for reporting diagnostic accuracy studies. BMJ.

[16] Balkenhol M (2018). H&E stain augmentation improves generalization of convolutional networks for histopathological mitosis detection: Medical Imaging 2018. Digital Pathology.

[17] Russakovsky O (2015). ImageNet large scale visual recognition challenge. Int. J. Comput. Vis..

[18] Simonyan, K. & Zisserman, A. Very Deep Convolutional Networks for Large-Scale Image Recognition. In International Conference on Learning Representations, 2015.

[19] He, K., Zhang, X., Ren, S. & Sun, J. Deep Residual Learning for Image Recognition. In Conference on Computer Vision and Pattern Recognition, 2016.

[20] Tan, M. & Le, Q. V. EfficientNet: Rethinking Model Scaling for Convolutional Neural Networks. In International Conference on Machine Learning, 2019.

[21] Thorsson V (2018). The Immune Landscape of Cancer. Immunity.

[22] Chattopadhyay, A., Sarkar, A., Howlader, P. & Balasubramanian, V. N. Grad-cam++: Generalized gradient-based visual explanations for deep convolutional networks. In 2018 IEEE Winter Conference on Applications of Computer Vision.

[23] Simonyan, K., Vedaldi, A. & Zisserman, A. Deep Inside Convolutional Networks: Visualising Image Classification Models and Saliency Maps. In International Conference on Learning Representations, 2014.

[24] Abadi, M. *et al*. TensorFlow: Large-scale machine learning on heterogeneous systems, 2015. Software available from tensorflow.org.

[25] Robin X (2011). pROC: an open-source package for R and S+ to analyze and compare ROC curves. BMC Bioinformatics.

[26] R Core Team (2021). R: A language and environment for statistical computing. R Foundation for Statistical Computing, Vienna, Austria. Available online at https://www.R-project.org/.

[27] Keisen. tf-keras-vis. https://github.com/keisen/tf-keras-vis (2020).

[28] Muti, H. S. *et al.* Development and validation of deep learning classifiers to detect Epstein-Barr virus and microsatellite instability status in gastric cancer: a retrospective multicentre cohort study. *Lancet Digit. Health*(2021) [Epub ahead of print]. 10.1016/S2589-7500(21)00133-3 PMC8460994 34417147

[29] Kanavati F (2020). Weakly-supervised learning for lung carcinoma classification using deep learning. Sci. Rep..

[30] Martinez-Ciarpaglini, C. *et al*. Assessing molecular subtypes of gastric cancer: microsatellite unstable and Epstein-Barr virus subtypes. Methods for detection and clinical and pathological implications. *ESMO. Open* **4**, e000470(2019). 10.1136/esmoopen-2018-000470 PMC6555614 31231566

[31] Camargo MC (2014). Improved survival of gastric cancer with tumour Epstein-Barr virus positivity: an international pooled analysis. Gut.

[32] Ilse, M., Tomczak, J. M. & Welling, M. Attention-based deep multiple instance learning. In International Conference on Machine Learning 2132–2141 (2018).

