# Supplementary material for: Detecting immunotherapy-sensitive subtype in gastric cancer using histologic image-based deep learning
Source: Sci Rep. 2021 Nov 22;11:22636. doi: 10.1038/s41598-021-02168-4 (PMC8608814; doi:10.1038/s41598-021-02168-4)
Supplement: Supplementary file 1 — Supplementary Information. [file 41598_2021_2168_MOESM1_ESM.pdf]

Supplementary Fig. S1. Data augmentation by changing colors randomly.

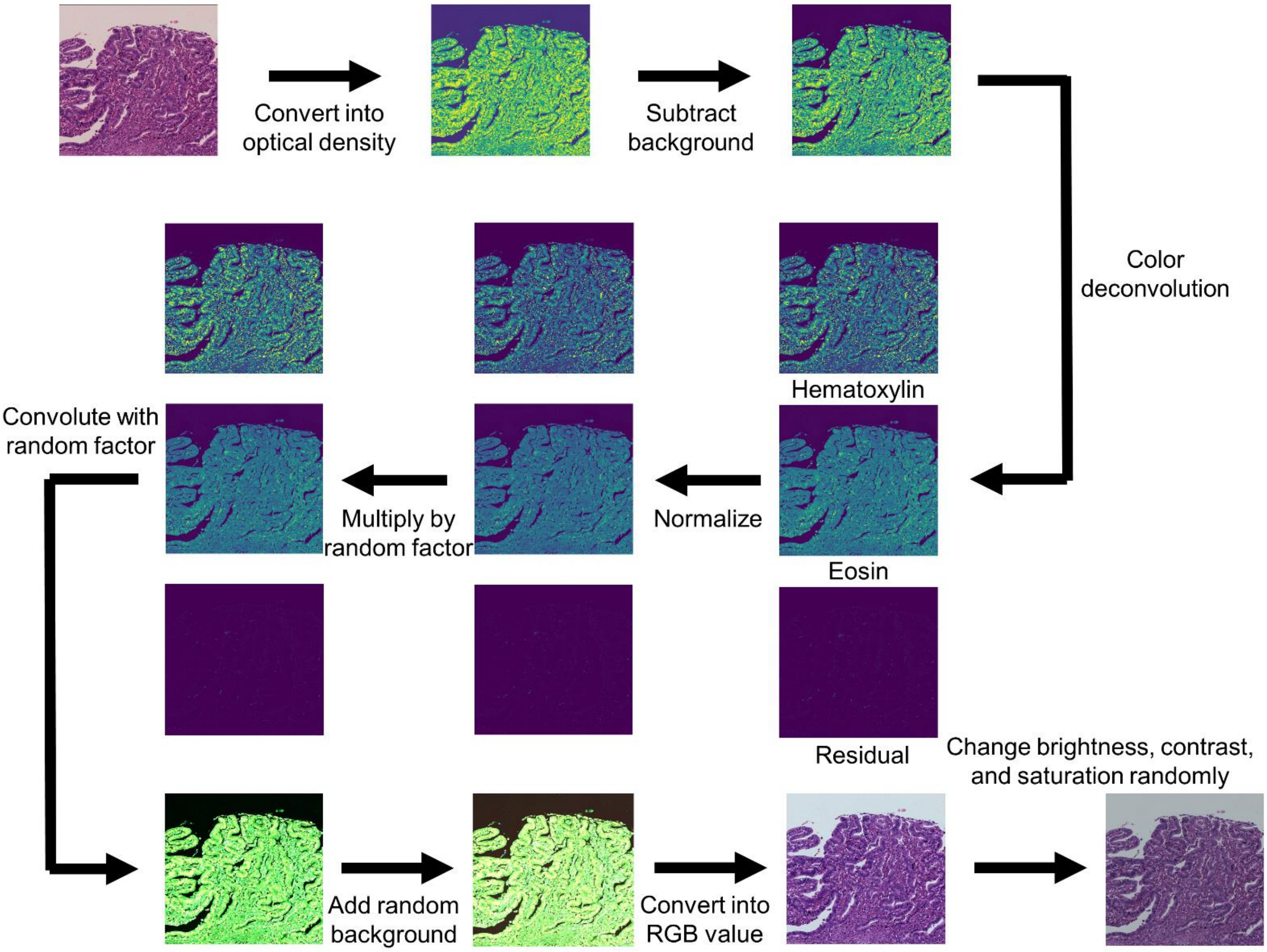

Supplementary Fig. S2. Architecture of deep learning models.

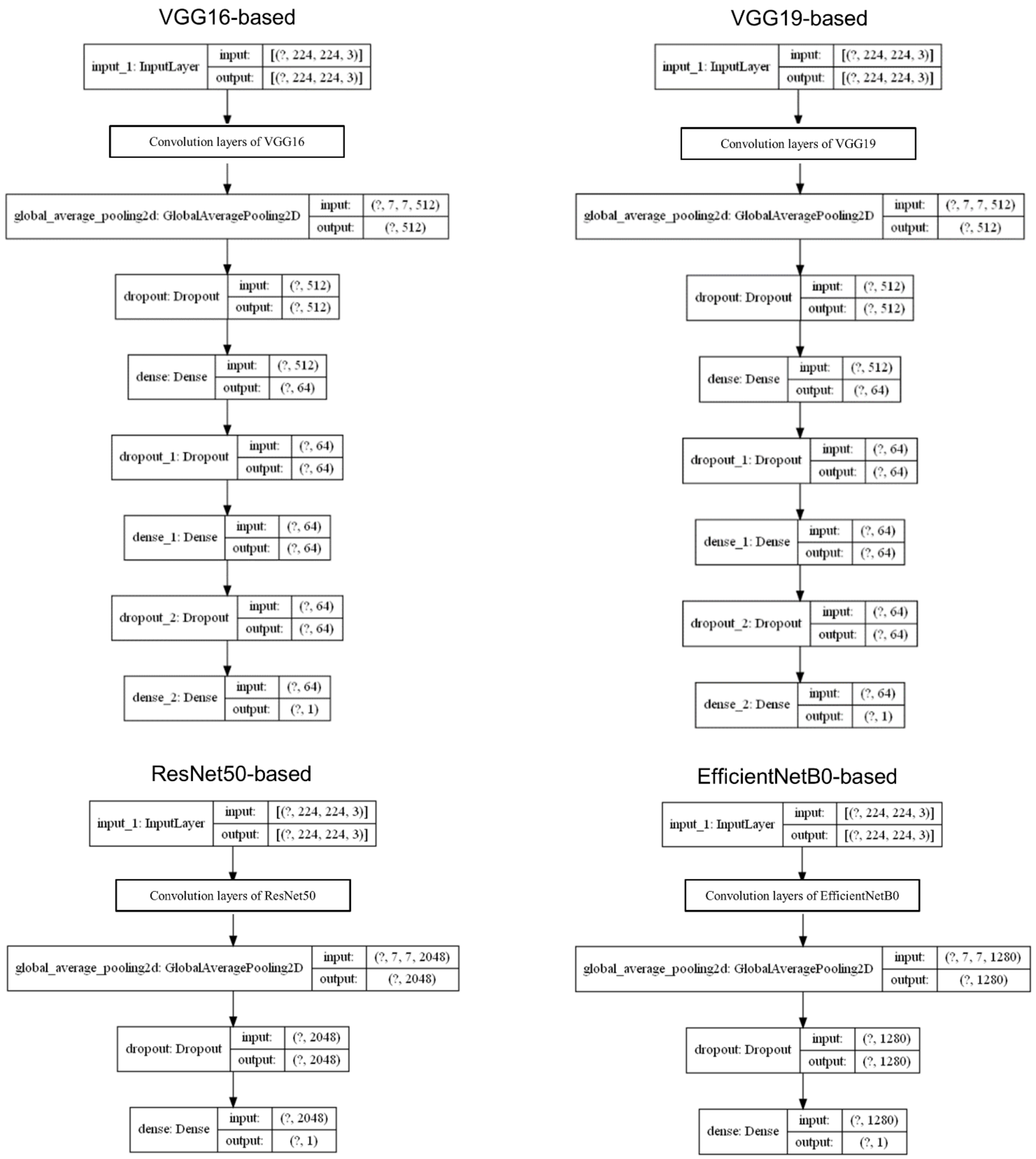

Supplementary Fig. S3. Tumor mutational burden of each subgroup. Patients were classified into EBV, MSI/dMMR, and others by the prediction of our deep learning model (a) or by the ground truth (b). *: p<0.001, Welch's t-test. EBV- Epstein-Barr virus, MSI- microsatellite instability, dMMR- mismatch repair deficiency.

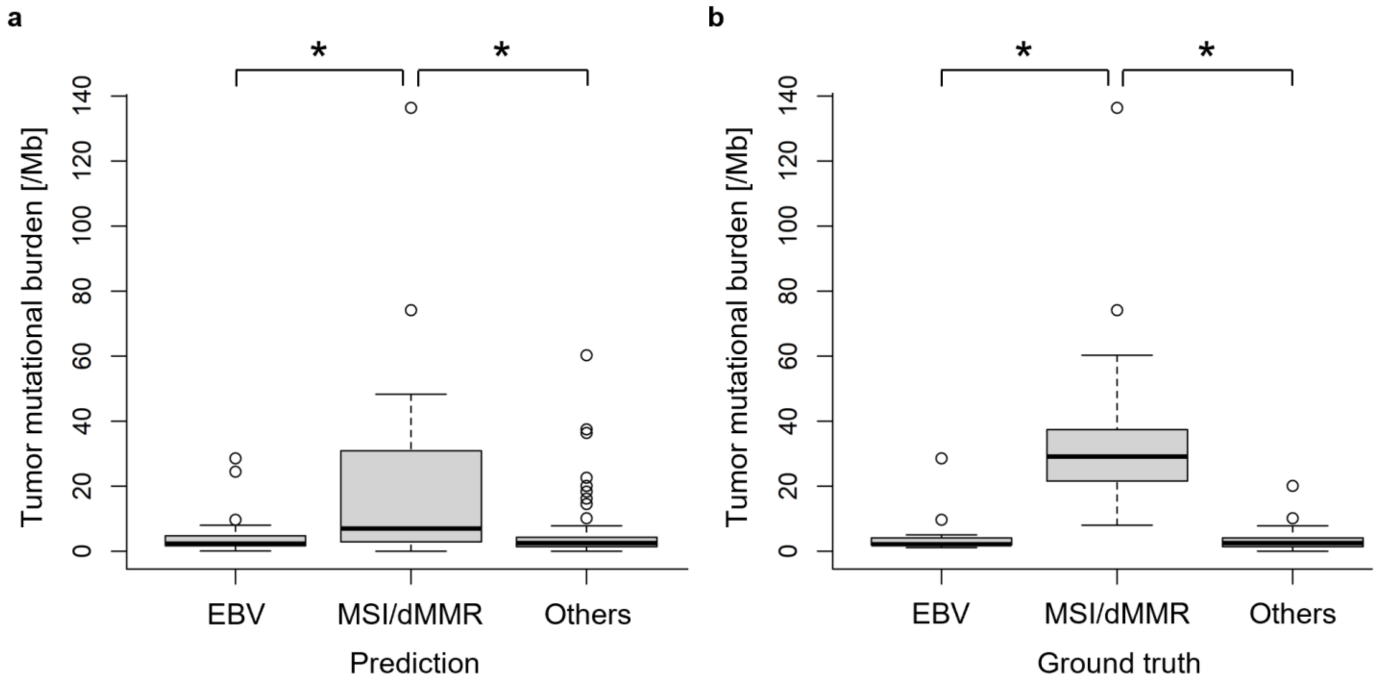

Supplementary Table S1. Checklist of STARD guideline.

Section & Topic	No	Item	Reported on page #
**TITLE OR ABSTRACT**			
	**1**	Identification as a study of diagnostic accuracy using at least one measure of accuracy<br>(such as sensitivity, specificity, predictive values, or AUC)	2
**ABSTRACT**			
	**2**	Structured summary of study design, methods, results, and conclusions<br>(for specific guidance, see STARD for Abstracts)	2-3
**INTRODUCTION**			
	**3**	Scientific and clinical background, including the intended use and clinical role of the index test	4-6
	**4**	Study objectives and hypotheses	6
**METHODS**			
Study design	**5**	Whether data collection was planned before the index test and reference standard<br>were performed (prospective study) or after (retrospective study)	6-8
Participants	**6**	Eligibility criteria	6-8
	**7**	On what basis potentially eligible participants were identified<br>(such as symptoms, results from previous tests, inclusion in registry)	6-8
	**8**	Where and when potentially eligible participants were identified (setting, location and dates)	6-8
	**9**	Whether participants formed a consecutive, random or convenience series	6-8
Test methods	**10a**	Index test, in sufficient detail to allow replication	8-13
	**10b**	Reference standard, in sufficient detail to allow replication	7-8
	**11**	Rationale for choosing the reference standard (if alternatives exist)	Not applicable
	**12a**	Definition of and rationale for test positivity cut-offs or result categories<br>of the index test, distinguishing pre-specified from exploratory	12
	**12b**	Definition of and rationale for test positivity cut-offs or result categories<br>of the reference standard, distinguishing pre-specified from exploratory	Not applicable
	**13a**	Whether clinical information and reference standard results were available<br>to the performers/readers of the index test	29
	**13b**	Whether clinical information and index test results were available	Not applicable

		to the assessors of the reference standard	
Analysis	**14**	Methods for estimating or comparing measures of diagnostic accuracy	12-13
	**15**	How indeterminate index test or reference standard results were handled	Not applicable
	**16**	How missing data on the index test and reference standard were handled	Not applicable
	**17**	Any analyses of variability in diagnostic accuracy, distinguishing pre-specified from exploratory	12-13
	**18**	Intended sample size and how it was determined	6-7
**RESULTS**			
Participants	**19**	Flow of participants, using a diagram	7-8, Fig.2
	**20**	Baseline demographic and clinical characteristics of participants	14, Table 1
	**21a**	Distribution of severity of disease in those with the target condition	14, Table 1
	**21b**	Distribution of alternative diagnoses in those without the target condition	14, Table 1
	**22**	Time interval and any clinical interventions between index test and reference standard	Not applicable
Test results	**23**	Cross tabulation of the index test results (or their distribution) by the results of the reference standard	24 (online data)
	**24**	Estimates of diagnostic accuracy and their precision (such as 95% confidence intervals)	15-16
	**25**	Any adverse events from performing the index test or the reference standard	Not applicable
**DISCUSSION**			
	**26**	Study limitations, including sources of potential bias, statistical uncertainty, and generalisability	23
	**27**	Implications for practice, including the intended use and clinical role of the index test	22
**OTHER INFORMATION**			
	**28**	Registration number and name of registry	Not applicable
	**29**	Where the full study protocol can be accessed	Not applicable
	**30**	Sources of funding and other support; role of funders	Not applicable

Supplementary Table S2. Hyperparameters used for training deep learning models.

	VGG16-based		VGG19-based		ResNet50-based		EfficientNetB0-based	
	Transfer learning	Successive fine tuning	Transfer learning	Successive fine tuning	Transfer learning	Successive fine tuning	Transfer learning	Successive fine tuning
Batch size	256	256	256	256	256	256	256	256
Optimizer	Adam	SGD	Adam	SGD	Adam	SGD	Adam	SGD
Learning rate	1.0×10^{-3}	2.0×10^{-5}	1.0×10^{-3}	2.0×10^{-5}	1.0×10^{-3}	2.0×10^{-5}	1.0×10^{-3}	1.0×10^{-4}
Cumulative total number of images used for training	1,280,000	3,584,000	1,280,000	4,275,200	512,000	3,584,000	512,000	5,990,400

Deep learning models were first trained with convolution layers frozen (transfer learning), and then trained entirely with small learning rate (successive fine tuning).

Supplementary Table S3. Pathological information of cohorts included in this study.

Parameters	TCGA (training)	TCGA (test)	TCGA (all)
Total number of samples	48	196	244
Molecular classification			
EBV	5 (10.4%)	18 (9.2%)	23 (9.4%)
MSI/dMMR	8 (16.7%)	36 (18.4%)	44 (18.0%)
Others	35 (72.9%)	142 (72.4%)	177 (72.5%)
CIN	25 (52.1%)	102 (52.0%)	127 (52.0%)
GS	10 (20.8%)	40 (20.4%)	50 (20.5%)
Lauren classification for EBV-negative and microsatellite stable tumors			
Intestinal	22 (62.9%)	92 (64.8%)	114 (64.4%)
Diffuse	10 (28.6%)	41 (28.9%)	51 (28.8%)
Mixed	3 (8.6%)	8 (5.6%)	11 (6.2%)
NA	0 (0.0%)	1 (0.7%)	1 (0.6%)
pT stage			
pT1	0 (0.0%)	9 (4.6%)	9 (3.7%)
pT2	6 (12.5%)	30 (15.3%)	36 (14.8%)
pT3	32 (66.7%)	110 (56.1%)	142 (58.2%)
pT4	10 (20.8%)	47 (24.0%)	57 (23.4%)

TCGA, The cancer genome atlas; EBV, Epstein-Barr virus; MSI, Microsatellite instability; dMMR, mismatch repair deficiency; CIN, chromosomal instable; GS, genomically stable; NA, not available.
